# *In vitro* breast cancer models for studying mechanisms of resistance to endocrine therapy

**DOI:** 10.37349/etat.2022.00084

**Published:** 2022-06-01

**Authors:** Gary J. Cheng, Euphemia Y. Leung, Dean C. Singleton

**Affiliations:** 1 Auckland Cancer Society Research Centre, Faculty of Medical and Health Sciences, The University of Auckland, Auckland 1023, New Zealand; 2 Maurice Wilkins Centre for Molecular Biodiscovery, The University of Auckland, Auckland 1023, New Zealand; 3 Department of Molecular Medicine and Pathology, The University of Auckland, Auckland 1023, New Zealand; University of Edinburgh, UK

**Keywords:** Endocrine therapy resistance, oestrogen receptor, breast cancer, cell line models

## Abstract

The development of endocrine resistance is a common reason for the failure of endocrine therapies in hormone receptor-positive breast cancer. This review provides an overview of the different types of *in vitro* models that have been developed as tools for studying endocrine resistance. *In vitro* models include cell lines that have been rendered endocrine-resistant by *ex vivo* treatment; cell lines with *de novo* resistance mechanisms, including genetic alterations; three-dimensional (3D) spheroid, co-culture, and mammosphere techniques; and patient-derived organoid models. In each case, the key discoveries, different analysis strategies that are suitable, and strengths and weaknesses are discussed. Certain recently developed methodologies that can be used to further characterize the biological changes involved in endocrine resistance are then emphasized, along with a commentary on the types of research outcomes that using these techniques can support. Finally, a discussion anticipates how these recent developments will shape future trends in the field. We hope this overview will serve as a useful resource for investigators that are interested in understanding and testing hypotheses related to mechanisms of endocrine therapy resistance.

## Introduction

Breast cancer is currently the most common cancer in women. It is estimated that 1 in 8 women will be diagnosed with breast cancer and 1 in 39 will die from it, ranking it first for total cancer cases in women both by incidence and mortality for the majority of countries in the world [1–3]. Breast cancers are a phenotypically diverse group of diseases and are usually divided into five surrogate intrinsic subtypes based on molecular characteristics of the presence or absence of oestrogen receptors (ERs), progesterone receptors (PRs), human epidermal growth factor receptor 2 (HER2), and Ki-67, a marker of proliferation (Table 1). Aberrations in cell signalling pathways are a fundamental process in the development and progression of cancer. Under normal circumstances, these receptors are involved in pathways that regulate cell growth, proliferation, differentiation, and survival, however, alterations in these pathways can result in uncontrolled growth and evasion of controlled cell death [4].

**Table 1.** Molecular subtypes of breast cancers and treatment options outside of traditional surgery and radiotherapy [5–10]

**Subtype**	**Molecular characteristics**	**Approximate incidence**	**Treatment options**
Luminal A-like	ER^+^, PR^±^, HER2^–^ Low Ki-67 Lower grade	60–70%	Endocrine therapy Targeted therapy Chemotherapy
Luminal B-like HER2^–^	ER^+^, PR^±^, HER2^–^ High Ki-67 Higher grade	10–20%	Endocrine therapy Targeted therapy Chemotherapy
Luminal B-like HER2^+^	ER^+^, PR^±^, HER2^+^ High Ki-67 Higher grade	13–15%	Endocrine therapy Targeted therapy Chemotherapy
HER2-enriched (non-luminal)	ER^–^, PR^–^, HER2^+^ High Ki-67 Higher grade		Chemotherapy Targeted therapy
Triple-negative	ER^–^, PR^–^, HER2^–^ High Ki-67 Higher grade	10–15%	Chemotherapy Targeted therapy Immunotherapy

Currently, the primary treatment methods for breast cancer are surgery, radiation therapy, chemotherapy, endocrine therapy, and targeted therapy [11]. Recent advances have also demonstrated the clinical benefit of immunotherapy, with immune checkpoint inhibitors gaining approval for the treatment of advanced triple receptor-negative breast cancer (TNBC) [12, 13]. Although early-stage localized breast cancers are effectively treated by breast-conserving surgery in conjunction with radiotherapy, patients who present with invasive disease have a much poorer prognosis [14]. Since oestrogen is one of the primary regulators of breast tissue growth by activating ER and resulting growth programs, targeting these pathways as a form of cancer therapy has revolutionized the treatment of ER^+^ breast cancer, which represent approximately 80% of cases [15–17].

Tamoxifen is a selective ER modulator (SERM) which modifies the transcriptional activity of ER by competitively binding to it to prevent activation by oestrogen and is one of the most commonly used front-line treatment methods for ER^+^ breast cancer [18]. However, despite initial clinical efficacy, the response is often temporary and approximately 40% of breast tumors will acquire resistance during treatment and ultimately relapse [19]. Furthermore, the mechanism of action of tamoxifen depends on the target tissue, tamoxifen can have either antagonistic or agonistic effects on ER [20, 21]. One of the most significant adverse effects of long-term tamoxifen treatment is the increased risk of endometrial cancer due to its agonistic effects, and therefore a more specific drug may be favorable [21, 22]. As drug resistance is often observed, second-line treatment options that target ER via different mechanisms are desired. Fulvestrant is currently an alternative drug approved for the treatment of ER^+^ breast cancer. It has a similar effect to tamoxifen, however, it is a pure ER antagonist with no agonistic effects [23]. Additionally, unlike tamoxifen, fulvestrant can induce the destabilization of ER as a whole and maintain suppression for prolonged periods of time and is thus classed as a selective ER degrader (SERD) [24].

Aromatase inhibitors (AIs) are the third class of drugs used as adjuvant therapy for breast cancer in postmenopausal women. Aromatase catalyzes the production of oestrogen in peripheral tissues such as adipose, skin, and breast. Thus, endocrine therapy with AIs acts by lowering oestrogen levels rather than by directly targeting ER as seen with SERMs and SERDs. Though adjuvant therapy with SERMs, SERDs and AIs decreases the risk of disease relapse, intrinsic or acquired resistance in patients does occur (collectively termed endocrine resistance). Therefore, there is an interest in developing methods and models to study and understand these mechanisms.

We provide this review of *in vitro* models as a resource for investigators that are interested in understanding and testing hypotheses related to mechanisms of endocrine therapy resistance. We highlight some of the methodologies that can be used to develop such models, provide an overview of the techniques applied in their characterization and discuss the types of outcomes that research using these models can support. We also emphasize some recent trends in the field and directions for future research.

## Hormone receptor-positive endocrine-resistant cell lines

Cell line models are reasonably homogeneous, inexpensive, and easy to propagate tools for researching breast cancer. Development of endocrine-resistant cell lines by exposing hormone receptor positive (HR^+^) cell lines to intermittent or increasing concentrations of SERM or SERD or by long-term oestrogen-deprivation (LTED) for several weeks to months in culture has been a widely employed technique over the last 40 years [25, 26]. Typically, the cultures undergo quiescence and cell death upon treatment, followed by eventual outgrowth of resistant clones over time, potentially representing a small subpopulation of pre-existing oestrogen-resistant cells within the initial culture [27]. Many investigators around the world have derived such models as tools to study endocrine therapy resistance mechanisms and to test interventions that seek to prevent or target this process. In addition to academic sources these models are also commercially available from various vendors and cell line collections [e.g., American Type Culture Collection (ATCC), European Collection of Authenticated Cell Cultures (ECACC), and Sigma-Aldrich], including MCF7 Tam1 (ATCC CRL-3435), tamoxifen-resistant 1 (TamR1, Sigma-Aldrich SCC101), TamR4 (ECACC 16022528), TamR7 (ECACC 16022509), and TamR8 (ECACC 16022510). Characterization of the changes that occur in the resistant cell lines has spanned various molecular techniques including genomic, transcriptomic, proteomic, metabolomic, and various other phenotypic assessments. While it is not possible to summarise all features of oestrogen-resistant HR^+^ models we will highlight some common characteristics in the following paragraphs, focusing on our own experience with these models, and emphasizing the changes that are most relevant to clinically observed mechanisms of resistance.

### Cell line models of tamoxifen resistance

**Figure 1.**
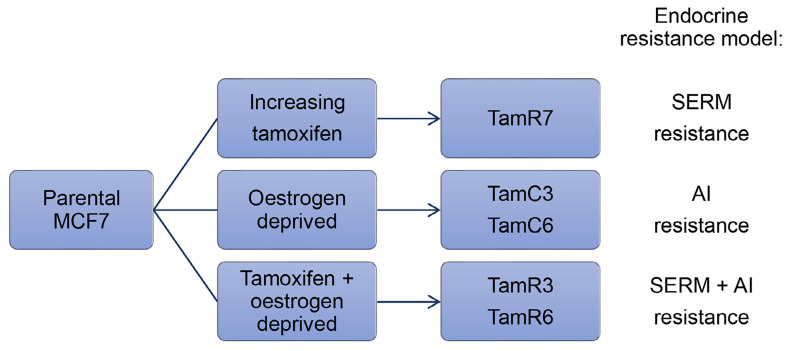
Summary of conditions used to generate endocrine therapy-resistant MCF7 sublines and the clinical relevance of the models

MCF7 cells are the most widely utilized breast cancer cell line in the world due to their high expression of ER, which closely mimics ER^+^ breast cancers [28]. Many of the studies regarding hormone therapy resistance have focused on MCF7. Leung et al. [29] showed that heterogeneity existed within the cell line by generating a number of tamoxifen-resistant MCF7 sublines via three differing conditions (Figure 1). Firstly, MCF7 cells were cultured in the presence of increasing concentrations of tamoxifen in a standard growth medium containing oestrogen to produce the TamR7 subline [29]. Secondly, prolonged culturing of MCF7 cells in the absence of oestrogen via phenol red-free medium supplemented with dextran-charcoal-treated fetal bovine serum (FBS; i.e. LTED), produced TamC3 and TamC6 sublines [29]. Finally, cells cultured in a combination of the above conditions, an oestrogen-free medium with the presence of tamoxifen, yielded TamR3 and TamR6 sublines [29]. It is important to note that all these conditions mimic situations under which clinical endocrine therapy resistance develops (i.e. SERM, AI, or SERM + AI combination treatment, see Figure 1) [29]. Although the resultant sublines displayed phenotypic heterogeneity all were resistant to tamoxifen irrespective of how they were derived [29]. Data characterizing DNA content, modal cell volume, and proliferation rate in the sublines suggested that the resistant cells were pre-existing within the population and were expanded under selective conditions [29–31].

Investigation via immunoblotting demonstrated that the sublines had altered levels of ER expression [29]. Although MCF7 is an ER^+^ cell line, ER expression in the parental line was lower when compared to the endocrine-resistant sublines with the exception of TamC6, which had comparable levels of expression to the parental line [29, 30]. The expression of HER2 also varied across the sublines. HER2 expression was unchanged in TamR7, extremely low in the TamC6 and TamR6 sublines, and was higher in the TamC3 and TamR3 sublines when compared to the parental line [29].

The endocrine-resistant sublines displayed alterations in proliferation and survival signaling pathways compared to the parental MCF7 cells. Five notable changes were observed: 1) increased levels of the phospho-extracellular signal-regulated kinase (ERK) in TamR7, TamC6, and TamR6; 2) decreased levels of phospho-protein kinase B (AKT) and phospho-p70 S6 kinase (p70S6K) in TamC3 and TamR3; 3) increased levels of phospho-epidermal growth factor receptor (EGFR) in response to tamoxifen treatment in TamC3 and TamR6; 4) decreased HER2 expression in TamC6 and TamR6; and 5) the presence of paired box 2 (PAX2) in TamC3, TamR3, TamC6 and TamR6 [29].

LTED sublines that were grown in the absence of oestrogen, without exposure to tamoxifen, were also tamoxifen-resistant, suggesting that cross-resistance was associated with dysfunction in common signaling pathways [29]. Response of these sublines to rapamycin, the compound from which everolimus was derived, showed that all variants were resistant, with the exception of TamR7 [29]. Furthermore, the addition of tamoxifen with rapamycin had no significant effect on TamC3, TamR3, and TamR6 even though the drug combination was able to cause robust dephosphorylation of p70S6K, suggesting that pathways other than phosphatidylinositol 3-kinase (PI3K)/AKT/mammalian target of rapamycin (mTOR) were utilized for survival [29]. Similarly, most of the endocrine therapy-resistant MCF7 sublines did not show sensitivity during treatment with PI3K inhibitors BEZ235 and GSK2126458 even though both drugs significantly inhibited AKT phosphorylation, with the exception of TamR7 which was sensitive to both BEZ235 and GSK2126458 [32].

Long-term exposure to tamoxifen to develop tamoxifen-resistant models has also been reported for other HR^+^ breast cancer cell lines, including T47D and BT474 [33]. Furthermore, a tamoxifen-resistant cell line model of breast invasive lobular carcinoma (ILC) has also been developed [34]. SUM44 cells were treated with tamoxifen until the resistant SUM44/LCCTam subline was derived. SUM44/LCCTam displayed decreased expression of ERα but increased expression of the oestrogen-related receptor γ (ERRγ), which was demonstrated to contribute to tamoxifen resistance.

### Cell line models of fulvestrant resistance

**Figure 2.**
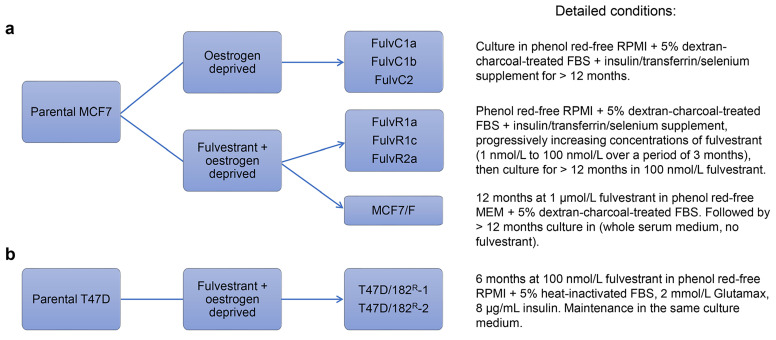
Summary of conditions used to generate fulvestrant-resistant MCF7 (a) and T47D (b) sublines by Liu et al. [35], Leung et al. [31] and Kirkegaard et al. [36]. MEM: minimal essential medium; RPMI: Roswell Park Memorial Institute 1640 culture medium

Though fulvestrant provides some benefits over tamoxifen, drug resistance also occurs. Liu et al. [35] developed fulvestrant-resistant MCF7 sublines (MCF7/F) by growing cells in the presence of fulvestrant and absence of oestrogen for a prolonged period (> 12 months). In contrast to the tamoxifen-resistant MCF7 cells described above, long-term exposure to fulvestrant resulted in MCF7 sublines that grew independently of oestrogen [35]. Resistant sublines did not express ER even after prolonged fulvestrant withdrawal (> 1 year), resulting in cells that had irreversibly lost ER expression and become hormone-independent [35]. Leung et al. [31] were able to reproduce the results of ER loss in the fulvestrant-resistant cell lines that were generated under similar conditions (FulvR1a, FulvR1c, and FulvR2a, see Figure 2a) and further extended these findings by showing that these resistant sublines had differing ploidy and mean cell volume, suggesting that pre-existing fulvestrant-resistant subpopulations existed within the culture before treatment [31]. Immunoblotting showed that sublines selected with fulvestrant lacked ER and PR expression, however, HER2 expression remained unchanged [31].

A loss of ER expression is sometimes correlated with an increase in the expression of EGFR [35, 37]. Liu et al. [35] found that EGFR expression was upregulated at both the messenger RNA (mRNA) and protein levels in MCF7/F cells. The EGFR inhibitor gefitinib was able to inhibit MCF7/F cell growth in a concentration-dependent manner, yet it had no effect on the growth of parental MCF7 cells [35]. This suggested that fulvestrant-resistant MCF7 cells acquired resistance by switching from ER-dependence to EGFR-dependence. Increased sensitivity of MCF7/F to U0126, a dual specificity mitogen-activated protein kinase kinase 1/2 (MEK1/2) inhibitor, and LY294002, a PI3K inhibitor, suggested that resistant sublines relied more on signaling via the mitogen-activated protein kinase (MAPK) and AKT pathway [35]. Similar results were also found by Leung et al. [31], whereby treatment of resistant sublines with BEZ235 and everolimus efficiently inhibited proliferation.

T47D is another cell line that is often used to represent luminal A breast cancer as it is ER^+^ and hormone-dependent [38]. Although the T47D and MCF7 cell lines initially seem very similar, studies have shown that there are some key differences between the lines such as the presence of high levels of mutant p53 in T47D making them more resistant to apoptosis, whereas MCF7 express wild-type and functional p53 [39]. Differences like this make T47D another valuable asset in the research of endocrine therapy resistance.

Kirkegaard et al. [36] developed fulvestrant-resistant T47D cell lines by growing them in the presence of fulvestrant for prolonged periods of time (approximately 6 months) without the removal of oestrogen from the growth media, resulting in two resistant sublines: T47D/182^R^-1 and T47D/182^R^-2 (Figure 2b). Similar to fulvestrant-resistant MCF7 lines, the resultant sublines also had a permanent loss of ER expression [36]. However, in contrast to MCF7, T47D sublines displayed significantly increased HER2 expression and decreased levels of EGFR and HER4 [36]. Although HER2 was upregulated in resistant cells, the levels of phosphorylated HER2 were similar to that of the parental cells [36]. Furthermore, treatment with AG825, a selective HER2 inhibitor, did not preferentially inhibit the growth of resistant cells, and knockdown of HER2 had no effect on growth in either parental or fulvestrant-resistant cells, suggesting that upregulation of the HER2 pathway was not the primary driver of fulvestrant-resistant survival upon loss of ER [36]. Further investigation showed that resistant sublines had high levels of proto-oncogene tyrosine-protein kinase Src (SRC), a tyrosine kinase that is crucial in the activation and dimerization of HER2 [36, 40]. Treatment with the SRC inhibitor dasatinib resulted in preferential growth inhibition of resistant sublines, confirming altered SRC signaling as a contributor to the mechanism of resistance [36].

### Cell line models of AI resistance

Because AIs act systemically it is difficult to model their pharmacology *in vitro*. One approach to this challenge has been the LTED strategy discussed above. Early use of LTED in T47D, ZR-75-1, and subclones of ZR-75-1 demonstrated a clonal pattern of development of oestrogen-independent growth, yet oestrogen responsiveness and sensitivity to tamoxifen were maintained, demonstrating retention of functional ER signaling [26]. In the case of ZR-75-1 cells, the oestrogen-independent clones within the parental cultures were estimated at a frequency of 1 in 1,000 cells. Notably, the investigators highlighted the predictable time course of events observed with both T47D and ZR-75-1 when developing endocrine-resistant models using this approach.

In addition to the cell lines already mentioned, LTED has been employed to develop HCC-1428 and MDA-MB-361 endocrine-resistant models [41]. Notably, many ER^+^ cell lines, particularly MCF7, express low levels of aromatase. Thus, more accurate modeling of AI pharmacology in cell lines has been achieved by overexpressing aromatase in MCF7, T47D, and ZR-75-1 [42].

### Cell line models with primary endocrine therapy resistance

While laboratory-developed models that have undergone treatment for extended periods of time are widely used to study endocrine therapy resistance, it is worth noting that some untreated HR^+^ breast cancer cell lines harbor genetic features that support “primary” endocrine therapy resistance. For example, the CAMA1 cell line harbors a fibroblast growth factor receptor 1 (*FGFR1*)/cyclin D1 (*CCND1*) co-amplification rendering it resistant to oestrogen deprivation but sensitive to inhibitors of FGFR1 and cyclin-dependent kinase 4/6 (CDK4/6) [43]. Thus, cell line models that “match” the genomic features of some patients can recapitulate the biology of tumors that carry molecular changes associated with poor responses to anti-oestrogen therapy without needing to be selected by *in vitro* treatment with SERM, SERD, or LTED.

### Generation of endocrine therapy-resistant cell line models by transfection of mutant oestrogen receptor 1 expression constructs

Missense mutations in oestrogen receptor 1 (*ESR1*; encoding ERα) most commonly affect residues in the oestrogen ligand-binding domain and adjacent regions, decreasing the responsiveness of ER to the ligand and enabling constitutive transcriptional activity [44]. Mutations in *ESR1* are a key mechanism of endocrine therapy resistance reported in approximately 30% (12–54%) of HR^+^ metastatic breast cancer patients [44]. *ESR1* mutations occur at a higher frequency in metastatic compared with primary breast tumors, implicating them as a cause of treatment failure. Indeed, mutant *ESR1* was detected in the circulating tumor DNA (ctDNA) of approximately 14% of HR^+^ breast cancer patients during disease progression [45]. The presence of ctDNA *ESR1* mutation was associated with an inferior response to AI therapy. Notably, these patients had all been treated with AI in the metastatic setting, implicating the selection of pre-existing *ESR1* mutant clones upon treatment in patients harboring extensive disease.

The most common missense changes in ERα are Y537N/C/S and D538G. Modeling *ESR1* mutant HR^+^ BC can be achieved by transfecting or transducing cell lines with *ESR1* mutant expression constructs, including Y537 and D538 mutants and ESR1-yes-associated protein 1 (YAP1) fusion genes [46, 47]. These systems impart features of endocrine resistance with modestly decreased sensitivity to tamoxifen/4-hydroxytamoxifen (4-OHT) and fulvestrant [46, 48].

*De novo ESR1* mutations (Y537C and Y537S) have also been described in endocrine-resistant cell lines generated by LTED [49]. In that work, the mutations were detected with variant allele frequency (VAF) of 30–50% in a proportion of LTED batches. In contrast, the parental cell lines did not contain detectable *ESR1* mutations, except for a single batch of SUM44 cells where *ESR1* Y537S mutation was detected at 0.001% VAF. These observations suggest that the selection pressure conferred by oestrogen deprivation promotes the enrichment of rare naturally occurring *ESR1* mutant clones within the culture.

### Generation of endocrine therapy-resistant cell line models by genome editing

Clustered regularly interspaced short palindromic repeat (CRISPR)-associated protein 9 (Cas9) genome editing has been used to generate a single allele knock-in MCF7 cell line model of the *ESR1* Y537S mutant [50]. This model demonstrated oestrogen-independence and decreased sensitivity to 4-OHT but retained responsiveness to fulvestrant *in vitro* and *in vivo*. Importantly, the model has enabled -omics studies, including RNA sequencing (RNA-seq) and ER chromatin immunoprecipitation followed by next-generation sequencing (ChIP-seq), demonstrating the power of genome editing in the development of endocrine-resistant cell line models. The investigators showed that recruitment of the basal transcription factor transcription factor IIH (TFIIH) by ERα Y537S and CDK7-dependent activation could be targeted by CDK inhibitors as a strategy for preventing the transcriptional activity of ER Y537S.

A model of ERα D538G was also generated by CRISPR-Cas9 genome editing and was used in addition to a Y537S mutant cell line to validate the pharmacodynamic effects of targeted ER degradation using novel proteolysis targeting chimeras (PROTACs) [51]. These reports highlight the power of CRISPR-Cas9 as a technique for rapidly generating cell line models with genomic alterations observed in clinical specimens that have progressed on endocrine therapy.

### Spheroid and mammosphere models

Three-dimensional (3D) culture of cell lines provides new possibilities for the development of more physiologically relevant human cancer models compared with 2D monolayer culture. Typical spheroids (i.e. 3D culture of cell lines) are grown in a low attachment culture vessel using a standard culture medium that includes FBS, often with the addition of a reconstituted basement membrane matrix (e.g., Matrigel) to aid spheroid development. The structure of spheroids provides a better model of the microenvironmental features of tumors including nutrient, pH, and oxygen gradients. Consequently, large spheroids display proliferating cells at the spheroid periphery, necrosis in the spheroid core, and a hypoxic region in the intermediate zone. The phenotypic heterogeneity associated with these stresses influences radiotherapy and chemotherapy response. MCF7 spheroids have been employed to study changes in cancer stem cell biology in response to chemotherapy [52]. Similarly, large T47D spheroids displayed decreased ER expression and resistance to 4-OHT [53]. Other advantages over 2D methods have been reported. For example, epigenetic changes identified by high-throughput chromosome conformation capture (Hi-C) profiling of breast cancer spheroids identified 3D-growth-specific chromatin interactions in endocrine-resistant models [54].

The mammosphere formation assay, another 3D technique that uses cell lines, is a commonly used *in vitro* method to quantify cancer-initiating stem cells that can propagate clonally to form spheres in free-floating conditions [55]. The mammosphere assay utilizes a culture medium that lacks FBS and reconstituted basement membrane matrix but includes supplementation with human epidermal growth factor, human basic fibroblast growth factor, and B27 supplement. This assay can investigate the breast cancer stem-like cell response to treatments [52, 56].

### Patient-derived organoid models

Breast cancer patient-derived organoids (PDOs) are a more recent development produced using primary cancer cells obtained from surgical resections [57, 58]. PDOs as a translational tool have revolutionized the study of breast cancer and therapy response tailored to the individual [59]. PDOs recapitulate the histological and genetic status of the original tumor, largely because the PDO models are cultured relatively short-term and in the presence of a serum-free culture medium compared to cell line models [60].

PDO pharmaco-phenotyping reflects the previous treatment responses of the corresponding donor patient. Thus, clinical patterns of sensitivity to tamoxifen were typically matched in the PDOs suggesting that resistance features that occur in patients are retained *ex vivo*, increasing the certainty that the resistance processes modeled in PDOs are relevant to clinical mechanisms [59].

As a living biobank, PDO models have the potential to be an effective platform for evaluating patient-specific drug sensitivity *in vitro*, which can prospectively guide treatment decisions for cancer patients at the terminal stage [61]. It also provides an alternative to reduce the use of animals in pre-clinical research [62]. Recently, a detailed optimized and versatile protocol for the long-term culture of PDOs to establish biobanked samples was published [63]. The methods include genetic manipulation, organoid selection, clonal culture, and orthotopic organoid transplantation in mice.

There are several other advantages of PDOs compared with traditional cell line models. Most prominently they retain greater levels of cellular heterogeneity, self-renewal/differentiation programs, 3D tissue morphology and are amendable to many high-throughput automation techniques, unlike patient-derived xenograft (PDX) models. In contrast to PDOs, cell lines that have been maintained in culture for many years or decades have evolved both genetically and non-genetically from their original biology, largely in response to culture conditions, resulting in changes to many phenotypes including morphology, proliferation rate, and drug response and discordance in behavior between laboratories [64].

The limitations of setting up PDO banks include the difficulties in scale-up and higher costs than traditional cell lines, including costs associated with the continual need for rigid extracellular matrix components. Notably, the types of scaffolding used for generating organoids can affect the drug test outcome [65, 66]. As PDOs display cellular heterogeneity due to the heterogeneous nature of breast cancer, their abilities to retain the mixed cellular organization and overall population phenotype over prolonged periods need to be considered [67, 68]. The ethical challenges arising from organoid use relate to the type of consent request from the patients, the privacy of cell donors, use of gene editing, issues of equity in the resulting treatment, commercialization, and long-term storage of biobanks [69].

While PDOs recapitulate many features with greater faithfulness than traditional cell line models, some features are poorly modeled. For example, the metabolic/nutrient environment within the tumor is unlikely to be well represented by the culture medium formulations used for the establishment and propagation of PDOs. In contrast, the development of a human plasma-like medium has enabled cell culture studies that are more physiologically relevant. This advance was used to demonstrate the artefactual effects that traditional culture medium formulations can have on gene dependency using CRISPR-Cas9 knockout (KO) functional genomic screens [70]. Thus, further advances in this area are needed to increase the relevance of PDOs, especially where they are being used in metabolic research projects. In addition to this limitation, a precise definition of breast cancer organoid culture medium components is lacking and will be required to better preserve donor tumor features [71].

A recent publication detailed the methodology for developing murine mammary organoids from both normal and tumor tissue [72]. This advance will encourage investigators to establish more elaborate genetically-engineered mouse models (GEMMs) that mirror the combinations of gene mutations observed in human tumors. These GEMMs can be studied *in vivo*, as well as utilized to generate organoids that can facilitate the deeper molecular analysis and interrogation that can only be achieved using *in vitro* cultures.

### PDX models and their use for generating early-passage *in vitro* organoids

While the focus of this review is on *in vitro* models, we do draw the reader’s attention to a few of the key advances in the area of PDX development and implications for *in vitro* breast cancer models. Many initial efforts to develop ER^+^ PDX models suffered from low tumor take rates, with reported engraftment rates of < 1–26% [73]. More recently, breast cancer PDX models that preserve patient intratumoral heterogeneity and clonal architecture have been reported, including endocrine therapy-resistant models that feature ERα Y537 mutations, *ESR1* gene amplification, or *ESR1*/*YAP1* fusion [47, 74].

The recent advances in PDX development, including better surgical techniques and mice with more optimal immunocompromised features, have improved the success rate. For example, prolactin-humanized NOD-*scid* IL2Rg^null^ (NSG) mice, termed NSG-Pro, demonstrate improved performance with both cell line xenografts (CLXs) and PDX models and allow a more faithful model of the clinical pattern of tumor response to tamoxifen and eventual development of tamoxifen resistance when compared with NSG mice [75].

These advances in PDX methodology will aid in the subsequent generation of early-passage PDOs that retain features of heterogeneity and can then be utilized in *in vitro* high-throughput analysis pipelines, including functional genomics and drug screens [63].

### Co-culture systems to model endocrine therapy resistance

While recent attention has focused on the development of PDO models, additional work has sought to develop co-culture systems that allow investigations of the direct (physical) interactions between cancer cells and stromal cells, or indirect interactions mediated by secretion of cell-derived molecules. For example, the co-culture of MCF7 cells with macrophages decreased the antitumor effects of oestrogen withdrawal, tamoxifen, or fulvestrant, providing an additional technique for developing cell culture models of endocrine therapy resistance [76]. Similarly, 3D co-culture of cell lines or PDOs in the extracellular matrix, with or without bone marrow stromal cells, to induce hormone independence has been used to recapitulate features of metastatic tumor growth within the bone [77]. Co-cultures of breast cancer cells with various other cell types including fibroblasts, adipocytes, muscle, endothelial and immune cells will facilitate the development of more relevant models for studying endocrine therapy resistance mechanisms.

## Molecular characterization of *in vitro* models of endocrine therapy resistance

The *in vitro* models introduced above have many advantages, including lower costs and fewer ethical implications, compared with *in vivo* models. A key limitation of cell line models is their high degree of homogeneity that results from long-term adaptation to cell culture conditions. While this homogeneity limits clinical relevance, it does allow for clearer experimental outcomes when studying the complex changes in transcriptional, genetic, epigenetic, and metabolic biology that support endocrine therapy resistance without the increased variability that comes with greater cellular heterogeneity. In contrast, the short-term nature of PDOs allows them to retain greater levels of tumor heterogeneity but this may create challenges for certain analysis techniques. In the following sections, we focus on different approaches that have been utilized to characterize *in vitro* models of endocrine therapy resistance, highlighting certain biological findings that are relevant to our understanding of this process in the clinic.

### Drug sensitivity screening and development of combination approaches

Once endocrine therapy-resistant cell line or PDO models have been generated, a common first step has been to evaluate their response to SERMs and SERDs, as well as larger more diverse compound libraries, including anticancer drug panels. A common finding is that LTED models, particularly those derived from MCF7 and HCC-1428, often retain or acquire increased levels of ER expression that permit ER-dependent, but oestrogen-independent cell growth, rendering them resistant to SERMs but sensitive to SERDs. This is consistent with clinical observations, loss of ER expression during endocrine resistance is only observed in a minor proportion of recurrent HR^+^/HER2^–^ tumors [78].

Tamoxifen-resistant sublines have been reported to display decreased sensitivity to DNA damaging chemotherapy [33, 79]. Similarly, fulvestrant-resistant sublines were used to understand the effects of the triple combination of fulvestrant, CDK4/6 inhibitor, and AKT inhibitor as a therapeutic approach for disease that has developed resistance to dual fulvestrant plus CDK4/6 inhibitor therapy [80]. This study provides important translational motivation for investigating AKT inhibition in patients that are progressing on endocrine therapy + CDK4/6 inhibitor combination regimens.

### Changes in ER expression and response to oestrogen

The increased expression of ER in some LTED cell lines permits oestrogen-independent ER transcriptional activity when oestrogen is deprived, but this adaptation can also cause growth-suppressive effects upon subsequent exposure to 17β-oestradiol (E2) [81]. Early studies using MCF7 cells showed that adaptation to LTED resulted in increased sensitivity to E2 [82]. This effect was reversible and LTED MCF7 cultures returned to a regular growth medium (containing 0.1–1 nmol/L E2) reacquired the level of oestrogen sensitivity observed in the parental MCF7 cells, suggesting that effects are not dependent on the selection of certain clones within the culture. Follow-up studies highlighted the activation of both extrinsic [first apoptosis signal receptor (Fas)-dependent] and intrinsic modes of apoptosis during E2-stimulated cell death in LTED or SERM-resistant MCF7 cells [83–85]. These key observations made using *in vitro* analysis explain the paradoxical inhibitory effect of E2 on LTED cells. The implications of these discoveries (apoptotic sensitivity resulting from oestrogen-deprivation upon re-exposure to E2) also provide a biological foundation for the reduction in breast cancer risk in women that start hormone replacement therapy long after menopause and the improvements in mortality due to long-term adjuvant tamoxifen therapy [86].

In contrast to MCF7, LTED cultures derived from MDA-MB-361 and ZR-75-1 were reported to decrease ER expression [87]. Thus, different changes in ER expression during endocrine resistance are observed in different cell lines and influence cellular response to E2.

### Transcriptional events

ER signaling in the nucleus depends on binding to specific DNA sequences (oestrogen response elements) and interaction with various other transcriptional co-activators. It is common to investigate ER transcriptional activity in endocrine therapy-resistant models by quantifying expression levels of ER responsive transcripts [e.g., trefoil factor 1 (*TFF1*), amphiregulin (*AREG*), PDZ domain protein kidney 1 (*PDZK1*)]. Yet, these assays only provide limited insight into the more widespread alterations in transcription that underlie endocrine therapy resistance (reviewed by Dittmer [88]). Transcriptome-level changes in gene expression have been reported using gene expression microarray or RNA-seq methods and can be compared with a similar analysis of clinical transcriptomic datasets to validate their relevance [89]. This type of analysis is useful for identifying cellular alterations that can produce resistance, in addition to effects on ER activity.

Of the many transcription factor-driven changes identified in cell line models, a key discovery has been the role of pioneer transcription factors, including forkhead box protein A1 (FOXA1), as mediators of resistance [90]. Pioneer transcription factors open inaccessible chromatin and enable ER to bind DNA. Loss of ER activity during therapy can result in a switch to other lineage-specific transcription factors including Notch [89, 91], activator protein-1 (AP-1) family of transcription factors [92] and stress-responsive transcription factors, including X-box binding protein-1 (XBP-1) [93] and hypoxia-inducible factor-1 (HIF-1) [94, 95]. The recognition that HIF-1 can compensate for ER suggests that increasing the relevance of *in vitro* models of endocrine resistance may be achieved by culturing cells under hypoxic conditions. While this can be accomplished by short-term hypoxic exposure we have developed a small series of breast cancer cell lines, including one that is HR^+^/HER2^+^, using continuous long-term culture in 5% O_2_ as an approach to derive more relevant cell line models [96].

In contrast to the various transcription factors that can compensate for ER, the E-twenty-six (ETS)-related transcription factor Elf5 has been demonstrated to suppress FOXA1- and ER-dependent oestrogen responsive programs and promote the acquisition of a basal phenotype [97]. Thus, changes in transcriptional programs that are acquired during endocrine resistance are an important consideration in characterizing *in vitro* cell lines and PDO models. Techniques to assess changes in the transcriptome including RNA-seq and gene expression microarrays provide valuable tools to profile these effects.

In addition to transcription factors and epigenetic regulators, certain long non-coding RNAs (lncRNAs) have been shown to influence the activity of endocrine therapy, suggesting that non-coding RNA species may be factors in the development of endocrine-resistant breast cancer [98]. For example, HOX antisense intergenic RNA (*HOTAIR*) is a lncRNA that is upregulated in tamoxifen-resistant disease [99]. Mechanistically, *HOTAIR* expression is directly repressed by ER. Upon ER antagonism the increased *HOTAIR* increases ER protein level and chromatin occupancy resulting in hormone-independence. Thymopoietin antisense transcript 1 (*TMPO-AS1*) is an oestrogen-inducible lncRNA that undergoes upregulation in LTED and 4-OHT-resistant MCF7 cells [100]. *TMPO-AS1* was found to stabilize *ESR1* mRNA and, like *HOTAIR*, supports the acquisition of endocrine resistance. In contrast, the ADAM metallopeptidase with thrombospondin type 1 motif 9 antisense RNA 2 (*ADAMTS9-AS2*) lncRNA was downregulated in tamoxifen-resistant MCF7 cells and lowly expressed in higher grade breast cancers [101]. *ADAMTS9-AS2* was found to antagonize microRNA-130a-5p which targets phosphatase and tensin homologue deleted on chromosome ten (PTEN) expression. Thus, loss of *ADAMTS9-AS2* expression contributes to tamoxifen resistance by derepressing microRNA-130a-5p resulting in PTEN downregulation.

### Protein expression-based profiling

ER transcriptional activity induces a number of mitogenic growth factors resulting in MAPK pathway stimulation, as well as mediators of the cell cycle including *MYC* and *CCND1*. Both the MAPK and PI3K/AKT/mTOR pathways act to sustain ligand-independent ER activity, and therefore represent key contributors to endocrine resistance and are frequent pathways for interrogation in endocrine-resistant models (reviewed by Hanker et al. [102]). The PI3K/AKT/mTOR signalling pathway is particularly relevant due to its central role in cell growth, proliferation, survival, motility, metabolism, and immune response [103, 104]. Alterations and hyperactivation of the PI3K/AKT/mTOR pathway are observed in many cancers, with estimations that aberrations are present in 60–70% of breast cancers [103, 105, 106]. The observation that somatic alterations in receptor tyrosine kinase (RTK; e.g., HER, FGFR receptors), RAS [e.g., neurofibromatosis type 1 (NF1)] and PI3K/AKT/mTOR (e.g., AKT1) pathways occur at higher frequency in endocrine resistant/metastatic disease compared with primary disease highlight the importance of these growth processes in endocrine resistance [102].

**Figure 3.**
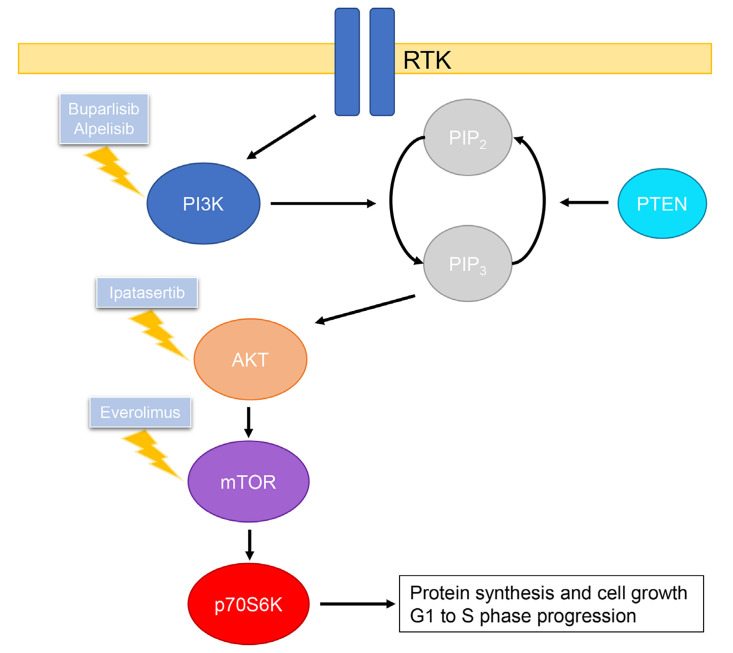
Summary of PI3K/AKT/mTOR pathway and drugs, including those under clinical investigation, that inhibit relevant targets

Class I PI3Ks are most frequently implicated in tumor transformation and growth [104]. Class I PI3Ks consist of two subunits, a p85 regulatory subunit and a p110 catalytic subunit [104]. Activation of PI3K is mediated by RTKs and ERs, which frees the p110 subunit allowing catalytic conversion of phosphatidylinositol 4,5-bisphosphate (PIP_2_) to phosphatidylinositol 3,4,5-triphosphate (PIP_3_) [103, 104]. This in turn leads to the phosphorylation and activation of AKT, and phosphorylated AKT activates mTOR, which further activates p70S6K which plays an essential role in the G1 to S phase progression [106–108]. This pathway is regulated by PTEN, which has inhibitory effects by dephosphorylating PIP_3_ to PIP_2_ (Figure 3) [106].

*PIK3CA*, the gene which encodes the p110α subunit of PI3Kα is found to be mutated in approximately 30% of all breast cancers, resulting in increased retention at the plasma membrane and increased catalytic activity [104, 109, 110]. Another driver of breast cancer is the loss of *PTEN* resulting in increased levels of phosphorylated AKT [104]. Together these mutations can lead to hyperactivity of the PI3K/AKT/mTOR pathway, which is a mechanism of acquired resistance to LTED as shown in several preclinical studies [111, 112].

As mutations in this pathway play such a central role in breast cancer, it is no surprise that it is a target of interest for therapies. Currently, there are a range of drugs in clinical trials that target various parts of the PI3K/AKT/mTOR pathway, however, only few are approved for the treatment of breast cancer [113]. Furthermore, inhibitors of the PI3K/AKT/mTOR pathway show minimal efficacy when used as a monotherapy, and thus these drugs are administered in conjunction with existing therapies such as tamoxifen or fulvestrant to increase antitumor efficacy.

Pan-PI3K inhibitors act by inhibiting the catalytic activity of all four isoforms of PI3K [113]. However, as they inhibit a wider range of targets there is an increased risk of toxicities, thus there are currently no pan-PI3K inhibitors approved for the treatment of breast cancer [113]. Buparlisib is the most extensively studied pan-PI3K inhibitor. In phase III BELLE-2 clinical trial, buparlisib in combination with fulvestrant was able to prolong progression-free survival by 1.9 months compared to fulvestrant alone in patients with advanced breast cancer [113, 114]. However, the questionable safety profile of this combination has prevented further testing.

Alpelisib is the only selective PI3Kα inhibitor currently approved by the Food and Drug Administration (FDA) for the treatment of advanced breast cancer [115]. Selective PI3Kα inhibitors provide benefits of having better efficacy and reduced risk of off-target toxicities in patients with *PIK3CA* mutations. In the SOLAR-1 phase III clinical trial, results showed that alpelisib administered in combination with fulvestrant was able to improve patient survival by up to 7.9 months compared to fulvestrant alone in *PIK3CA* mutated patients [116].

Targeting AKT prevents the activation of mTOR. Ipatasertib is a competitive inhibitor of all three isoforms of AKT and is currently in phase II and III trials for the treatment of advanced breast cancer [113, 117]. The phase II LOTUS study in patients with TNBC showed that ipatasertib in combination with paclitaxel was able to increase progression-free survival by 1.3 months compared to the paclitaxel plus placebo group [118].

As mTOR forms the catalytic subunit of two complexes, mTOR complex 1 (mTORC1) and mTORC2, competitive inhibitors of mTOR disrupt the downstream effects of cell growth, proliferation, and survival mediated by both complexes [103, 113]. In contrast, everolimus is an allosteric inhibitor of mTORC1 and is currently approved by the FDA for the treatment of metastatic breast cancer [119]. The phase III BOLERO-2 study involving patients with HER2-negative breast cancer showed that everolimus in combination with exemestane led to significantly better progression-free survival by approximately 4 months [119]. Another phase II clinical study (TAMRAD) with tamoxifen in combination with everolimus showed a 46% reduction in risk of progression compared to tamoxifen alone [120].

The clinical development of PI3K/AKT/mTOR targeted therapies for breast cancer treatment and the increasing appreciation that PI3K/AKT/mTOR contributes to endocrine resistance emphasises the relevance of studying the pathway in cell line or PDO models. For example, the importance of investigating the PI3K/AKT/mTOR pathway was highlighted in studies of endocrine-resistant MCF7 cells generated by introduction of erb-b2 receptor tyrosine kinase 2 (*ERBB2*; HER2) activating missense mutations [121]. While *ERBB2* activating mutations are uncommon in primary breast cancer they are enriched in recurrent tumors. Gain of this alteration caused hyperactivation of the PI3K/AKT/mTOR pathway that could be suppressed by everolimus. Thus, the various processes that enable endocrine resistance often converge on the PI3K/AKT/mTOR pathway, making it a sensible pathway for investigation when endocrine resistance is observed.

While many researchers focus on a small number of proteins (e.g., ER, PR, HER2, RTK/MAPK, and PI3K/AKT/mTOR pathway proteins) others have used proteomic analysis to quantify changes at the proteome scale. For example, analysis of MCF7-TamR cells demonstrated altered expression of > 600 proteins, with notable increased expression of S100P [122]. S100P was shown to confer resistance to tamoxifen when overexpressed in parental MCF7 cells, suggesting it represents a potential resistance mechanism. Of note, trophoblast cell surface antigen 2 (TROP2) expression was induced in TamR cells potentially indicating an opportunity for targeting by TROP2 antibody drug conjugates, for example, sacituzumab govitecan an FDA-approved third-line treatment for metastatic TNBC [123]. The induction of TROP2 expression in other endocrine-resistant models needs to be confirmed to validate this finding.

In the setting of ILC, the Wnt ligand WNT4 was found to be directly regulated by ER and was involved in oestrogen-dependent cell proliferation [124]. Upon development of oestrogen-independence (ILC-LTED), WNT4 expression was maintained by activated nuclear factor kappa-B (NF-κB) signalling and acted to suppress p21 expression. Thus, investigating Wnt and NF-κB can aid in the characterisation of endocrine-resistant models, especially for ILC.

### Mutational profiling

The identification and monitoring of *ESR1* missense mutations using ctDNA has highlighted their potential role in endocrine resistance. A common technique for detecting ctDNA is droplet digital PCR (ddPCR). ddPCR can be applied to detect and monitor changes in *ESR1* mutation abundance at low VAF in *in vitro* models of endocrine resistance [49].

Large scale efforts to catalogue the somatic mutational landscape of endocrine therapy-resistant disease using whole genome sequencing have been conducted (reviewed by Hanker et al. [102]). These studies have generated a short list of genes with most encoding proteins involved in RTK-MAPK, PI3K, or nuclear functions. Assessment of these genetic alterations may aid in the characterization of *in vitro* models of endocrine resistance.

### Epigenetic changes

Cell lines have been vital tools in defining the altered patterns of DNA methylation and chromatin modification and structure that are associated with endocrine therapy resistance [54, 89, 125]. These epigenetic events underscore phenotypic changes. For example, the activation of cholesterol biosynthesis due to increased histone 3 lysine 27 acetylation (H3K27ac) at regulatory regions of cholesterol biosynthesis acts as a mechanism of resistance to AI [126]. ChIP-seq was used to demonstrate that H3K27ac-enriched enhancers marked phenotypic clones that underwent expansion during disease progression [127]. Interaction of ER with these enhancers was stabilized by transcription factor Yin Yang 1 (YY1). These observations demonstrate the important effects that epigenetic clonal diversity can have in contributing to tumor evolution and represent biology that warrants further investigation.

### Functional genomics

Many functional genomic studies have investigated gene dependency in breast cancer cell lines, including by both RNA interference (RNAi)- and CRISPR-Cas9 KO-based methodologies [128, 129]. Several studies utilized kinome-wide small interfering RNA (siRNA) screens to identify kinases that may be implicated in endocrine therapy resistance by comparing effects in LTED cells with their parental cell line. These studies highlighted increased dependencies on insulin receptor/insulin-like growth factor-1 receptor, polo-like kinase 1, and the SRC family kinase LYN in LTED cells [41, 130, 131]. In addition, key insights into the regulation of early 2 factor (E2F) by ER were discovered following siRNA screens that identified CDK4 [87]. The resulting focus on CDK4/6 inhibitors in this disease setting led to subsequent FDA approvals of palbociclib, ribociclib, and abemaciclib in patients with advanced HR^+^/HER2^–^ breast cancer. Ultimately, these focused RNAi screens have provided important insights into the crosstalk between kinase signaling and the oestrogen-independent transcriptional activity of ER.

More recently, whole-genome CRISPR-Cas9 KO screens were used to identify genes responsible for cellular response to either 4-OHT or fulvestrant [132, 133]. These studies identified an important role for the mammalian switch/sucrose non-fermentable (SWI/SNF) nucleosome remodeling complex, particularly the AT-rich interacting domain-containing protein 1A gene (ARID1A) subunit, in response to therapy, with loss of *ARID1A* resulting in endocrine therapy resistance. *ARID1A* KO in cell lines or *ARID1A* mutation in patient tumors was associated with changes in biology towards a basal-like phenotype and decreased sensitivity to SERDs [133]. Further studies demonstrated that ARID1A was required for FOXA1-dependent genome-wide localization of ER and suppression of ER-dependent transcription. Notably, the loss of ARID1A in *ARID1A* mutant cells alters the epigenetic state at ER-bound *cis*-regulatory elements resulting in increased levels of histone acetylation and recruitment of the bromodomain and extra-terminal motif (BET) bromodomain-containing protein 4 (BRD4) [132]. This altered epigenetics creates a reliance on BRD4 that results in increased sensitivity to BET bromodomain inhibitors.

Genome-wide CRISPR-Cas9 KO screens also identified C-terminal SRC kinase (CSK) as a key factor in endocrine therapy response [134]. CSK KO stimulated oestradiol-independent growth and enabled the growth of MCF7 xenografts in ovariectomized mice. A second screen was then conducted to identify the genes responsible for the oestradiol-independent growth provided by CSK KO. This secondary screen identified the p21 protein-activated kinase 2 (PAK2) and the adapter molecule c-crk (CRK) as essential genes in the CSK KO background. This study highlighted a previously underappreciated ER-dependent feedback mechanism, whereby loss of ER signaling that occurs during endocrine therapy decreases CSK levels causing derepression of SRC family kinase (SFK)- and PAK2-dependent oncogenic signaling pathways that support oestrogen independence.

These studies demonstrate the power of conducting unbiased whole-genome *in vitro* functional genomic screens to reveal biomarkers of therapy response and the application of these findings to assist in the interpretation of clinically annotated genomic datasets to generate a deeper understanding of disease mechanisms. Further functional genomic investigations using both improved screening libraries and techniques and more relevant disease models will provide an even greater understanding of these processes, as recently reviewed by Nguyen and Caldas [135].

### Single-cell analysis techniques

Because PDOs retain greater levels of heterogeneity they are particularly suited to studying the phenotypic diversity within tumors and how this diversity changes in response to therapy at cellular resolution using single-cell genomic analysis techniques, including single-cell RNA-seq (scRNA-seq) and mass cytometry/cytometry by time of flight (CyTOF) [136–139]. Furthermore, techniques like single-cell DNA sequencing (scDNA-seq) allow the clonal evolution and intratumoral genetic heterogeneity to be examined in these models [140]. Alterations in chromatin accessibility, which are particularly relevant to ER-dependent cell lineage/identity programs, can be studied by single-cell assay for transposase-accessible chromatin (ATAC) sequencing (scATAC-seq) and epigenomic changes can be studied using single-cell ChIP-seq (scChIP-seq) [141, 142].

Recent advances in spatial transcriptomics have expanded the possibilities of single-cell analysis to the use of histological tissue sections [143]. Spatial transcriptomic methods have detailed the tumor and immune cell interactions in HER2^+^ breast cancers [144]. This technology will also enable a tissue-level spatially resolved single-cell analysis of endocrine-resistant cell states. Further integration of this information with other tumor features will provide a clearer understanding of *in situ* tumor processes that cause endocrine resistance, including the effects of intratumoral heterogeneity.

Thus, newly developed single-cell techniques and more relevant models are providing a deeper understanding of the biology that underpins the eventual development of endocrine therapy resistance in patients with advanced breast cancer.

## Conclusions

**Figure 4.**
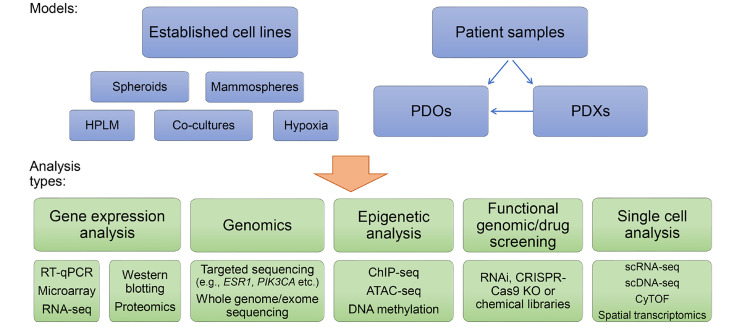
*In vitro* breast cancer endocrine resistance models and opportunities for their analysis. RT-qPCR: quantitative reverse transcription polymerase chain reaction; HPLM: human plasma-like medium

In this article, we have highlighted the important contributions that cell line models have had in the understanding of endocrine therapy responsiveness and resistance. The breadth of endocrine therapy-resistant models developed and characterized worldwide has benefitted the research community. In future, these tools will continue to be a mainstay of research, particularly in areas of biomarker discovery and validation and drug development. Furthermore, advances in genome editing will drive the continued development of more genetically relevant cell line models that replicate the most common genetic features of human breast cancers, increasing the suitability of these models. There has been a notable recent shift towards the development and use of PDOs in breast cancer research, including early-passage PDX-derived models. These models will play a significantly greater role in future studies in this area and are particularly suited to studies of intratumoral diversity and tumor evolution during therapy. Applying single-cell genomic techniques, including scRNA-seq, scDNA-seq, scATAC-seq, CyTOF, and spatial transcriptomics, to understand these processes at single-cell resolution will provide greater insight into the critical changes in biology that allow resistance to arise across different patients (Figure 4).

Of note, the immunotherapy revolution that is occurring in oncology and its interactions with endocrine therapy will continue to gain research attention in the near future. While *in vivo* models will play a large part in this work, *in vitro* systems to investigate cancer and immune cell interactions will also be needed. Combining these models and techniques with continue to drive discoveries that can be translated into the clinic to improve outcomes for patients with advanced therapy-resistant disease.

## Abbreviations

3D: three-dimensional
4-OHT: 4-hydroxytamoxifen
*ADAMTS9-AS2*: ADAM metallopeptidase with thrombospondin type 1 motif 9 antisense RNA 2
AIs: aromatase inhibitors
AKT: protein kinase B
ARID1A: AT-rich interacting domain-containing protein 1A gene
ATAC: assay for transposase-accessible chromatin
Cas9: clustered regularly interspaced short palindromic repeat-associated protein 9
CDK4/6: cyclin-dependent kinase 4/6
ChIP-seq: chromatin immunoprecipitation followed by next-generation sequencing
CRISPR: clustered regularly interspaced short palindromic repeat
CSK: C-terminal SRCproto-oncogene tyrosine-protein kinase Src kinase
ctDNA: circulating tumor DNA
E2: 17β-oestradiol
ECACC: European Collection of Authenticated Cell Cultures
EGFR: epidermal growth factor receptor
ERs: oestrogen receptors
*ESR1*: oestrogen receptor 1
FBS: fetal bovine serum
FOXA1: forkhead box protein A1
HER2: human epidermal growth factor receptor 2
*HOTAIR*: HOX antisense intergenic RNA
HR^+^: hormone receptor positive
ILC: invasive lobular carcinoma
KO: knockout
lncRNAs: long non-coding RNAs
LTED: long-term oestrogen-deprivation
MAPK: mitogen-activated protein kinase
MCF7/F: fulvestrant-resistant MCF7 subline
mTOR: mammalian target of rapamycin
mTORC1: mechanistic target of rapamycin complex 1
NSG: NOD-*scid* IL2Rg^null^
p70S6K: p70 S6 kinase
PDOs: patient-derived organoids
PDX: patient-derived xenograft
PI3K: phosphatidylinositol 3-kinase
PRs: progesterone receptors
PTEN: phosphatase and tensin homologue deleted on chromosome ten
RNA-seq: RNA sequencing
RTK: receptor tyrosine kinase
SERD: selective oestrogen receptor degrader
SERM: selective oestrogen receptor modulator
SRC: proto-oncogene tyrosine-protein kinase Src
TamR1: tamoxifen-resistant 1
TNBC: triple receptor-negative breast cancer
TROP2: trophoblast cell surface antigen 2
VAF: variant allele frequency

## Declarations

### Copyright

© The Author(s) 2022.
